# Modeling Functional Connectivity for Bears Among Spawning Salmon Waterways in Haíɫzaqv (Heiltsuk) Territory, Coastal British Columbia

**DOI:** 10.1002/ece3.71579

**Published:** 2025-07-14

**Authors:** Ilona Mihalik, Mathieu Bourbonnais, William Housty, Kevin Starr, Paul Paquet, Chris Darimont

**Affiliations:** ^1^ Department of Geography University of Victoria BC Canada; ^2^ Raincoast Conservation Foundation BC Canada; ^3^ Department of Earth, Environmental and Geographic Sciences University of British Columbia Okanagan BC Canada; ^4^ Heiltsuk Integrated Resource Management Department BC Canada

**Keywords:** circuitscape, codevelopment, corridors, Heiltsuk knowledge, Indigenous knowledge, land management, landscape connectivity, resource waves, spatiotemporal, Ursus

## Abstract

Understanding how functional connectivity can provide mobile consumers access to key resources can inform habitat management. The spatial arrangement of landscape features, for example, can affect movement among resource patches. Guided by the Haíɫzaqv (Heiltsuk) Integrated Resource Management Department (HIRMD), and within Haíɫzaqv Territory, coastal British Columbia (BC), Canada, our objectives were to (1) estimate functional connectivity for grizzly and black bears ( *Ursus arctos* and *U. americanus* , respectively) among aggregations of spawning Pacific salmon (*Oncorhynchus* spp.), (2) identify important movement pathways for landscape planning, and (3) contribute to the growing body of functional connectivity research on dynamic ecological systems. Using circuit theory and least cost paths, we predicted movement among salmon spawning reaches within a 5618 km^2^ study area. Variables affecting bear movement were parameterized by drawing on the relevant literature and Haíɫzaqv Knowledge. We validated our cumulative resistance surface with observed movements as identified via genetic recapture data. Modeled current from Circuitscape suggested areas of high connectivity between salmon spawns within and among watersheds. Our least cost paths model identified principal routes, which we then ranked to illustrate possible corridors for consideration by HIRMD planners. Understanding movement among salmon spawns, a fitness‐related food, provides key information to inform landscape planning for bears. Further, our work provides an example of connectivity research codeveloped, executed, and applied with an Indigenous government.

## Graphical abstract

Our team, which included the Haíɫzaqv (Heiltsuk) Integrated Resource Management Department (HIRMD), estimated functional connectivity for grizzly and black bears among aggregations of spawning Pacific salmon, a fitness‐related food. Our model, validated with movement data from genetic tagging, identified important movement pathways to inform landscape planning within Haíɫzaqv Territory. Our work offers an example of applied connectivity research co‐developed with an Indigenous government.

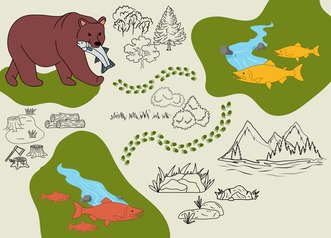

## 1 Introduction

A growing and worldwide threat to wildlife is the magnitude and rate of human landscape modification. Whereas the ecological implications are profound, the alteration of landscape connectivity—the extent to which movement among resources (e.g., food, water, breeding habitat, or partners) is facilitated or impeded on a landscape (Taylor et al. 1993; Cosgrove et al. 2018)—in particular, can pose multiple challenges for mobile consumers at varying spatial and temporal scales. Over shorter periods and distances, individuals can experience a reduction in access to important resources (Rudnick et al. 2012). Over longer periods and distances, disruptions to movement and dispersal can potentially impede gene flow. Owing to these realities, land managers (hereafter “managers”) face increasing pressure to retain and protect landscape connectivity for wildlife, particularly in environments with low levels of industrial development or disturbance (Betts et al. 2017; Ramírez‐Delgado et al. 2022). However, while a large body of connectivity literature exists, a major challenge is the effective translation of research findings into management potential that align with local contextual needs. Indeed, successful landscape connectivity planning and implementation requires long‐term collaboration between researchers, local and Indigenous governments, managers, partners, and other rightsholders involved (Keeley et al. 2018, 2019).

Across heterogeneous landscapes, mobile consumers must navigate natural and human‐caused barriers to exploit important resources. Natural features, such as rugged terrain, waterbodies, and glaciers, have always imposed barriers to movement (Lewis et al. 2015; Muñoz‐Mendoza et al. 2017; Paetkau et al. 1998). More recently, however, anthropogenic barriers from agriculture, resource extraction (e.g., logging, mining), and other forms of development have intensified (Venter et al. 2016), influencing the accessibility and availability of resources across space (Doherty et al. 2021). Consumers that rely on access to spatially and temporally variable resources, such as resource waves, are particularly impacted (Holdo et al. 2011; Seidler et al. 2015; Bolger et al. 2008). Within resource wave systems, temporally brief pulses of resources occur at varied locations across a landscape (e.g., plant green‐up moving from low to high elevations; Sawyer and Kauffman 2011; Armstrong et al. 2016). Although consumers may capitalize on short‐lived resources at individual locations, an emergent property of resource waves is the sustained energy provided over time to mobile consumers accessing multiple pulses (Armstrong et al. 2016).

Despite the growing risks to mobile wildlife that depend on resource phenology, research and planning relevant to landscape connectivity among resource pulses are relatively new. Recently, approaches for estimating landscape connectivity have expanded from measuring structural connectivity (i.e., physical and spatial components) to additionally consider functional connectivity, the aspect of landscape connectivity focusing on the perception and behavioral responses of organisms to landscape structure in navigating among resource patches (Tischendorf and Fahrig 2000; Cushman et al. 2006; Rudnick et al. 2012; Auffret et al. 2015). Several analytical tools now exist for estimating functional connectivity among habitat patches, protected areas, or individual occurrences (Cushman et al. 2013). Among those most commonly used, resistance‐based models predict movements of organisms based on user‐defined resistance surfaces, which are spatially explicit representations of landscape permeability to movement across environments (Zeller et al. 2012). However, few examples exist for predictions in resource wave contexts. Bishop‐Taylor et al. (2017) included remotely sensed time series imagery to account for seasonal variation in surface water habitats in southeastern Australia into a resistance layer for multiple water‐dependent migratory species. Additionally, early connectivity work using telemetry data combined separate sub‐models accounting for seasonal landscape changes within a resistance model to estimate movements by wolves ( *Canis lupus* ) in Banff National Park, Alberta (Paquet et al. 1996). Recent approaches for African elephants ( *Loxodonta africana* ) likewise incorporated telemetry data on movement patterns across seasons into resistance surfaces (Osipova et al. 2019; Kaszta et al. 2021). Building on these examples, we aim here to use resistance‐based modeling to predict the functional connectivity among spatial patterns of a resource wave system in an applied and collaborative case study.

Here, we examine functional connectivity for grizzly (náṇ in Haíɫzaqvḷa language; *Ursus arctos* ) and black bears (ƛ̓á; *U. americanus* ) in Haíɫzaqv Territory, coastal British Columbia (BC), Canada, in the context of a key food item that collectively offers a resource wave: Pacific salmon (miá; *Oncorhynchus* spp.). Five species of salmon inhabit the coastal waters of the region, each returning to spawn in freshwater systems at differing times from late April to late December. How far upstream salmon migrate to their respective reaches (i.e., spawning and rearing extent) also varies by species and location (Gende et al. 2002; Service et al. 2019). Accordingly, such spatial and temporal variation in spawning pulses creates the potential for prolonged access over the spawning season for consumers, such as bears. Most bears on the coast move to feed at spawning salmon congregations throughout midsummer and fall months (Mowat and Heard 2006; Hatler et al. 2008). Although consumption varies across individuals, salmon provides a major contribution to diet and the accumulation of fat reserves, and is associated with increased body size, mobility, cub litter size, reproductive success, and reduced stress hormones (Hilderbrand et al. 1999; Bryan et al. 2013). Accordingly, salmon availability is a primary driver of inter‐seasonal movements during the spawn; studies from Alaska have shown that brown bears track this resource wave, both among pulses within the same watershed or stream neighborhood (Wirsing et al. 2018; Deacy et al. 2019), and among larger watersheds (Barnes 1990; Schindler et al. 2013; Deacy et al. 2016). Further, recent dietary analyses suggested individuals that move among multiple spawns throughout the season benefit from increased consumption overall (Deacy et al. 2018). In coastal BC, black bears similarly benefit from access to multiple salmon spawns; individuals in watersheds that offered a more diverse suite of spawns consumed more salmon throughout the year (Service et al. 2019).

Although the importance of access to multiple salmon spawns for coastal grizzly and black bears is well‐known, to our knowledge, no studies have examined or predicted how landscape features might affect movements among spawns by bears. Working by invitation with the Haíɫzaqv Integrated Resource Management Department (HIRMD), with which we have worked for over a decade on wildlife projects (e.g., Adams et al. 2014; Housty et al. 2014; Service et al. 2014, 2019; Henson et al. 2021), our objectives were to (1) estimate functional connectivity among salmon reaches within and across watersheds for grizzly and black bears, and (2) identify particularly important routes, with emphasis on areas around and between some watersheds of importance to the Haíɫzaqv. HIRMD, which manages and implements forestry initiatives within their Territory, sought additional information on this culturally and ecologically important bear‐salmon system to complement their own Haíɫzaqv Knowledge and existing scientific information for consideration in their decision‐making and conservation planning. To support the first objective, we evaluated our modeled connectivity predictions by comparing the outputs with empirical data on individual bear detections within the same study area, hypothesizing that certain landscape features may influence movements by bears (details below). Lastly, our aim was to (3) contribute to the growing body of functional connectivity research on dynamic ecological systems. We note that we do not incorporate the temporal or within‐season dynamics of this resource wave system, but instead focus on the spatial dimension here as a preliminary first step.

## 2 Methods

### 2.1 Study Area

**FIGURE 1:**
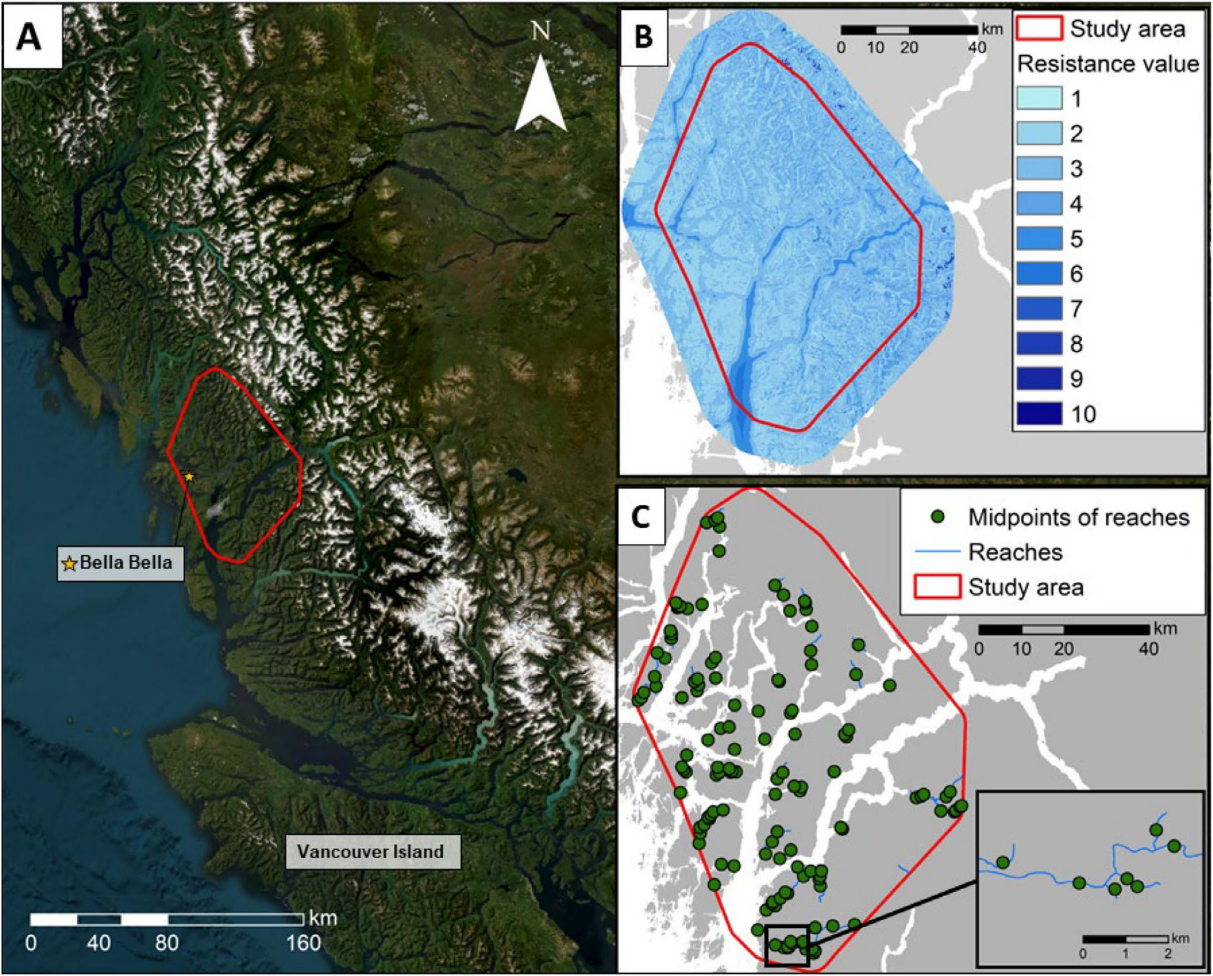
(A) Study area (red line) within Haíɫzaqv (Heiltsuk) Territory on the central coast of British Columbia, Canada. (B) Cumulative resistance surface created from three habitat variables: Landcover class, water, and terrain ruggedness. (C) Locations of the 134 midpoints of spawning salmon reaches used as focal nodes.

The study area is located on what is now known as the central coast of BC, Canada (Figure 1A). The area spans approximately 5618 km^2^ and is within the Territories of the Haíɫzaqv Nation. The landscape, described previously (Adams et al. 2017; Bryan et al. 2013; Service et al. 2014, 2019), includes mainland valleys, large inlets, and many coastal islands. The region is characterized by climatic, biological, and geographic variation, covering the Coastal Western Hemlock, Mountain Hemlock, and Coastal Mountain‐heather Alpine biogeoclimatic zones at different elevations (Meidinger and Pojar 1991). As the area is accessible only by boat or air, industrial disturbance is lower than many other landscapes at these latitudes. Indeed, a recent analysis that spanned most of the grizzly bear and Pacific salmon distribution in BC found that coastal watersheds had strikingly low levels (0.0 to 3.5) of Human Footprint Index (“HFI”; on 50‐point scale, with HFI = 50 representing urban environments; Adams et al. 2024). Additionally, although forestry activity is now present throughout, a relatively high proportion of intact forest remains (5.4% of forested area logged; Pearson 2010; DellaSala et al. 2011) and much of the area lacks connected road systems (Shackelford et al. 2018; Proctor et al. 2020). The area also has low human density since colonization and associated genocide imposed by settler Europeans (Boyd 1999).

### 2.2 Data Sources

All original spatial data are publicly available and were downloaded from the BC Data Catalog (https://catalogue.data.gov.bc.ca) in September 2022. We obtained landcover, water, and forestry harvest year data from the provincial 2021 Vegetation Resources Inventory (VRI). We reclassified landcover, water, and harvest data to represent our habitat variables (below). We derived the terrain ruggedness index from a digital elevation model (DEM) using the Raster Terrain Analysis plugin in QGIS (following methods from Riley et al. 1999). Finally, spatial data for Pacific salmon spawning zones were obtained from the BC Historical Fish Distribution layer.

### 2.3 Resistance Surface

#### 2.3.1 Selection of Habitat Variables

We selected variables likely to affect grizzly and black bear movement and scaled their resistance based on examination of related literature and our own local observations. We chose to combine information on both bear species in our model creation as we reasoned both black and grizzly bears had similar movement preferences and restrictions, and owing to the limited genetic data available from grizzly bears to use in our validation. Accordingly, we considered what features might influence landscape permeability and engaged as a team (comprising all authors of the study) in discussions that drew on local Haíɫzaqv Knowledge, relevant literature, and existing research observations in the area. Specifically and briefly, after codeveloping our research questions with William Housty (WH) and Kevin Starr (KS) of HIRMD to meet envisioned management goals, we collectively reviewed a draft analytical approach (Circuitscape and LCP; below) and candidate variables identified from the literature for further discussion. During our collaborative meetings, WH and KS provided their local knowledge and ecological geomatics expertise and drew on the context of forestry and land use planning within their Territory. The team also drew on considerable insight into bear movement patterns from previous demographic and mark–recapture work within the study area led by a Haíɫzaqv scientist and Knowledge Holder (Housty et al. 2014). In consensus after multiple online meetings, we selected three variables (Table 1) on which to focus a connectivity model. Further descriptions of the decision processes involved in parameterization and their justification are presented below.

**TABLE 1:** Assigned resistance values (unitless, ranging from 1 [very low] to 6 [very high]) for each habitat variable and corresponding class. Values were used to create the three habitat variable resistance layers for grizzly and black bears in Haíɫzaqv (Heiltsuk) Territory on the central coast of British Columbia, Canada.

Habitat variable	Resistance	Assigned resistance value
Terrain ruggedness (TRI)		
1–25	Very low	1
25–50	Low	2
50–88	Low–medium	3
88–139	Medium‐high	4
139–230	High	5
230–271	Very high	6
Landcover		
Mature forest (non‐harvested, or harvested over 75 years ago, or projected age over 75)	Very low	1
Bryoids, shrubland, herbs	Very low	1
Barren, exposed rock	Very low	1
Regenerating forest (harvested 10–75 years ago)	Low–medium	3
Snow, ice	Very high	6
Water by distance to shore (km)		
< 1	Low–medium	3
1–3	High	5
> 3	Very high	6

#### 2.3.2 Terrain Ruggedness

Although local knowledge from contemporary observations suggests that bears can occur at higher elevations in the area and often scale steep terrain, we assumed ruggedness would impose some resistance to movement during the salmon spawn. Over longer time periods, landscapes with higher ruggedness have been associated with a greater variance in genetic relatedness in grizzly bears (Lewis et al. 2015), suggesting ruggedness imposes costs to movement over the long term. Context also matters. Specifically, at a smaller temporal scale and in areas of increased human activity, grizzly bears have been found to prefer rugged and steep landscapes as they inhibit human access and development (Apps et al. 2004). However, in areas of lower human density, both bear species were found to prefer lower elevation valley bottoms during late summer and fall months before denning (Collins et al. 2005; McLellan and Hovey 2001; Sager‐Fradkin et al. 2008). This could be explained by the seasonal shift in their food sources to lower elevations and the lower energetic costs of traveling over flat terrain (Carnahan et al. 2021; Roever et al. 2008). Given these patterns and details of our study area above, we increased resistance values with terrain ruggedness at intervals following Jenks natural breaks (Table 1). This classification method splits data into clusters of similar (low variation) values, that is, the natural breaks within the dataset.

#### 2.3.3 Landcover Class

Coastal bears use a variety of landscape features, though some likely act as barriers or impediments to movement. Originally, we suggested separating landcover into seven classes, with distinct categories for different forest covers. In discussing the applicability to our study area with HIRMD, however, we instead combined the different forest covers into one class (“mature forest”) for unharvested and/or mature (> 75 years) coniferous, broadleaf, or mixed stands, reasoning that they pose similar resistance. Accordingly, we identified five landcover types that affect bear movement: (1) mature forest; (2) bryoids, shrubland, herbs; (3) barren, exposed rock; (4) regenerating forest; and (5) snow, ice. We assigned all landcover types, except regenerating forest and snow/ice, the lowest resistance score (Table 1). Both bear species have been found to select for habitats with mature forests and shrubs of mixed types and species, as well as avalanche chutes (Apps et al. 2004; McLellan and Hovey 2001; Nielsen et al. 2002, 2010; Hiller et al. 2015; Cushman et al. 2006; Belant et al. 2010). In addition, we assumed that bare rock environments, which grizzly bears select (Apps et al. 2004; Waller and Mace 1997; Zager et al. 1983), would not act as a barrier to their movement. We assigned regenerating stands between 10 and 75 years old a low–medium resistance value (Table 1). Discussions with HIRMD and previous literature identified that landscape permeability for bears likely varies based on post‐logging forest age class. Between 10 and 75 years after industrial harvest, for example, cut blocks in productive coastal temperate rainforests enter and progress through a hyper‐dense stage (“stem exclusion”), comprising small‐ and medium‐diameter trees (Wallmo and Schoen 1980; Wells 1996; Albert and Schoen 2013). Although this stage can last up to 150 years before self‐thinning occurs, it is especially prominent up to ~ 75 years (Wells 1996). We therefore expected that forest cover in this period would act as a moderate impediment to movement, and as a window during which few shrub and understory plant resources are available (Wallmo and Schoen 1980; Alaback 1982, 1984). Finally, we assigned snow and ice environments the highest resistance value as these landscapes provide few food resources and have been shown to be avoided by bears (Cushman et al. 2006; Berman 2019).

#### 2.3.4 Water

Bears can be proficient swimmers, but water likely imposes a greater cost than terrestrial movement at some distances. Grizzly bears have been recorded swimming 34 km to remote islands in Alaska (Mattson et al. 2005). Additionally, evidence suggests black bears (especially females) in southeastern Arkansas cross the White River (200 m wide) but are deflected by the Mississippi River (1600 m wide; White et al. 2000). Further, and although over a longer time period, Paetkau et al. (1998) suggested water crossings 2–4 km and > 7 km in width are barriers to brown bear dispersal based on the genetic isolation of populations within the Kodiak Archipelago, Alaska. Accordingly, we estimated that smaller crossings (< 1 km) such as small inlets, rivers, or lakes within our study area would impose moderate resistance, whereas those greater than 1 km would increasingly resist movement. We therefore assigned water the following resistance values, based on distance from land: < 1 km = low/medium, 1–3 km = high, and > 3 km = very high (Table 1) using the Multiple Ring Buffer Analysis tool in ArcMap 10.7.1 (ESRI 2019).

#### 2.3.5 Cumulative Layer

We summed the three individual resistance layers equally to identify areas of high and low cumulative resistance. The three layers were created using ArcMap 10.7.1 (ESRI 2019). We resampled each layer to a spatial resolution of 150 m to balance the computational capacity of running the models, while following similar scales from previous brown bear connectivity and landcover suitability modeling analyses (Almasieh et al. 2019; Falcucci et al. 2008; Henson et al. 2021). After combining the three layers into our cumulative (additive) layer, our new resistance scale ranged from 1 (low resistance) to 10 (high resistance). We then clipped the final layer to the study area with a 10 km buffer to account for potential edge effects (Koen et al. 2010; Figure 1B).

### 2.4 Focal Nodes

To model connectivity for bears among salmon spawning areas, both within and between watersheds, we used the midpoints of each Pacific salmon species reach (hereafter “reach”) per stream as focal nodes. Reaches varied in length, from 19 m to 11.3 km, with an average of 1.7 km. Streams were first distinguished by their “watershed code,” a numeric code unique to each watershed tributary. In many cases, species had multiple reaches within an individual stream. For these, we used the midpoint of all reaches belonging to an individual species per stream. In the cases where multiple reach‐midpoints overlapped, we used the mean center of nodes within 150 m of one another to simplify the midpoints, which corresponded with the 150 m cell resolution of our cumulative resistance layer. In total, we defined 134 focal nodes representing salmon reaches within our study area (Figure 1C).

### 2.5 Functional Connectivity

We used the Circuitscape package (v0.1.0; Anantharaman et al. 2019) in Julia and the leastcostpath package (v1.8.7; Lewis 2021) in R (v4.2.2; R Core Team 2022) to model functional connectivity and identify priority linkages between salmon hotspots. Drawing on the behavior of electrical circuits, Circuitscape applies circuit theory (McRae et al. 2008) in an ecological setting to predict connectivity. Circuitscape uses cumulative resistance and focal node raster or network files to generate current maps iteratively for each pair of nodes, with the additional option of generating a map of cumulative or maximum current. Current density (*I*, ampere) displayed in the maps represents the probability of a “random walker” passing through each cell, based on the resistance, as it moves between focal nodes (McRae et al. 2008; Shah and McRae 2008). Locations of high‐current density represent areas of higher predicted connectivity with few obstructions, while “pinch points” indicate particularly constricted areas of high current (McRae et al. 2008). We ran Circuitscape in pairwise mode to estimate connectivity iteratively between each pair of focal nodes (i.e., midpoints of reaches). Cells were connected to their eight immediate neighbors in calculations, both cardinal (first‐order) and diagonal (second‐order). Our surface contained 584,260 cells and current was calculated between 9045 focal node pairs.

To support the applied planning scenario envisioned by HIRMD, we additionally used Least Cost Paths (LCP) analysis to identify the most important pathways and potential candidate corridors from the Circuitscape cumulative current map. Whereas Circuitscape is a useful tool for predicting all possible movement pathways across the landscape, the output does not delineate specific corridors, which is often of importance to land managers and planners (Parrott et al. 2019). In contrast, LCP predicts only the least costly route between node pairs, rather than all possible movements. Across a raster surface, the LCP identifies the pathway with the lowest cost value summed across its cells (Cushman et al. 2013). Accordingly, we converted our current map output from Circuitscape to a conductance transition matrix and used the *create_FETE_lcps()* function within the leastcostpaths package in R to determine the pathways of the highest conductance between each node pair. We calculated the cost distance (i.e., accumulated cost) for each LCP, which we ranked further to determine the top 5% pathways of highest conductance and lowest cost. Finally, we considered the geometry of potential candidate corridors from the top pathways. In the literature, the recommended width for both bear species varies based on the level of industrial, residential, and recreational activity in the area. To allow for unrestricted movement of grizzly and black bears in areas that include recreational trails, Ford et al. (2020) suggested corridor widths of 1300 m and 1460 m, respectively, following a case study within the Bow Valley Provincial Park, Alberta. Considering the denser forest cover, lower accessibility and human density, and minimal recreational activity within our study area, as well as operational planning by HIRMD, we applied 1200 m buffers to the top pathways to illustrate candidate bear corridors to be considered in future decision‐making and modification by HIRMD. Although the output is not meant to be prescriptive and includes a conservative buffer width in comparison with recommendations for the Bow Valley, Alberta, our purpose was to highlight via visualization how corridors could be situated across this coastal landscape.

### 2.6 Validation of Cumulative Resistance Layer

Validating the accuracy of resistance parameters, particularly those derived from expert and local knowledge, is a crucial component for predicting connectivity (Laliberté and St‐Laurent 2020; Riordan‐Short et al. 2023). However, less than 6% of connectivity modeling publications include any validation (Creech et al. 2024). Numerous approaches and metrics exist for validating resistance layers and model outputs, with the decision over which to use dependent on the study context and availability of independent data (Wade et al. 2015; Zeller et al. 2018; Creech et al. 2024; Poor et al. 2024).

We used an independent genetic dataset of individual bears from the same study area to evaluate the ability of our resistance surface to accurately represent landscape resistance. Accordingly, we compared whether real‐world transits by bears, inferred from genotypes from grizzly and black bear hair collected at hair snag sites between 2015 and 2019, were associated with the effective conductance between sites predicted from Circuitscape using our cumulative resistance layer.

#### 2.6.1 Hair Snag Sample Collection and Identification of Bear Transits

Hair samples were collected noninvasively from snag sites (*n* = 66) baited with a non‐reward substance and evenly spaced throughout the study area (in variable habitats and including along salmon streams) as part of a multiyear bear monitoring project (e.g., Adams et al. 2017; Service et al. 2019). Baited snag sites were “revisited” twice, approximately 10–14 days apart, within a sampling season (early May to mid‐June) after setup each year. The sex, species, and individual identity were determined from the collected hair using information from seven microsatellite loci plus a sex marker (Wildlife Genetics International, Nelson, BC). Further information on these data and methods can be found in Bryan et al. (2013), Adams et al. (2017, 2024), and Service et al. (2014, 2019).

We created graphs (i.e., nodes and edges) using the genetically tagged hair samples from individual bears (*n* = 66 grizzly, 199 black bears) to identify transits between sites. Edge lists (pairs of site connections; Proulx et al. 2005) of transits were created and based on sites visited by individuals within a sampling season; for example, if an individual bear had visited two different sites within the same season, we assumed the individual transited between the two sites. Of all possible unique site pairs (*n* = 2145), 2091 were not transited (“no transit”) from 2015 to 2019, while 54 were transited (“transited”) at least once. Graphs were created using the igraph package (v1.4.2; Csardi and Nepusz 2006) in the R programming language (v4.2.2; R Core Team 2022).

#### 2.6.2 Comparison of Bear Transits With Effective Conductance From Circuitscape

We evaluated the validity of our cumulative resistance surface by examining whether the effective conductance calculated between each snag site pair was associated with the presence of a transit from the genetic data. Effective conductance (*Ĝ*), the inverse of effective resistance (*R̂*), is a measure of how easily current can flow between two focal nodes considering all pathways and resistors (McRae et al. 2008; Shah and McRae 2008). We ran Circuitscape with our cumulative resistance surface, using the hair snag sites as focal nodes, and converted the output (pairwise effective resistances) into effective conductance values; if our resistance surface generally aligned with landscape resistors to bear movement within the area, we predicted a positive association between the probability of transit and conductance, while accounting for distance between site pairs (reasoning that closer pairs would have a higher probability of transit). Accordingly, we used Firth's penalized‐likelihood logistic regression (Firth 1993), which reduces potential bias from unbalanced datasets, to evaluate whether the probability of transit (0/1) was associated with effective conductance. We additionally included the Euclidean distance between site pairs (ranging 1400–102,000 m) as an explanatory variable to account for the potential influence of distance among sites on the probability of transit.

## 3 Results

### 3.1 Functional Connectivity

**FIGURE 2:**
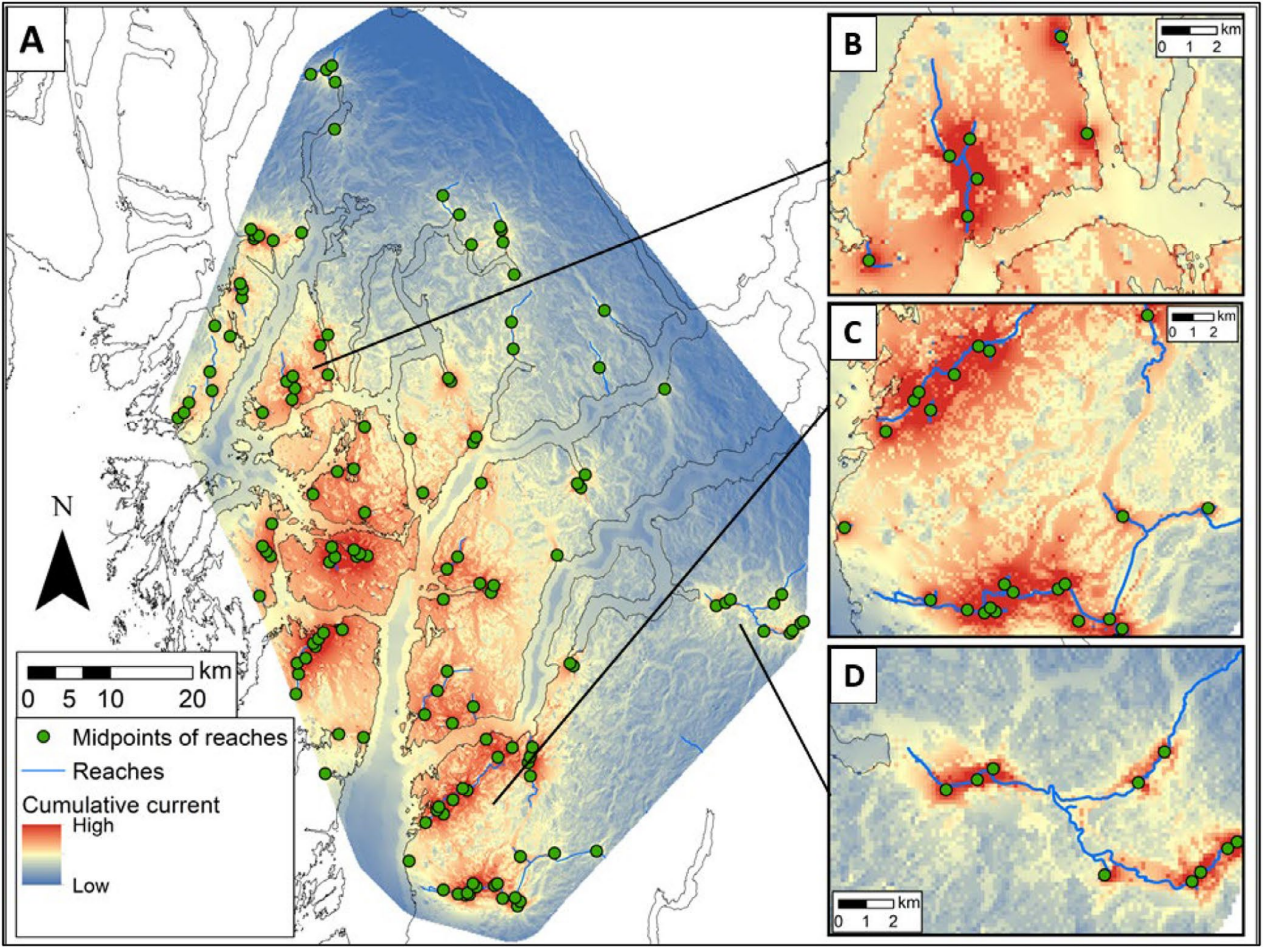
(A) Cumulative current map output using Circuitscape, which estimated connectivity for grizzly and black bears among midpoints of spawning salmon reaches in Haíɫzaqv (Heiltsuk) Territory on the central coast of British Columbia, Canada. Red zones depict dense areas of high predicted connectivity. Yellow/orange zones depict areas of constricted connectivity, referred to as ‘pinch points’. Predicted connectivity was highest (B) between and within watersheds throughout the western islands and (C) southern portion of the mainland. (D) The eastern half of the study area displayed areas of high predicted connectivity between reaches within watersheds. Maps displayed with percent clip stretch (2%).

Our estimation of cumulative current identified spatial patterns in predicted connectivity among salmon reaches at the inter‐watershed and intra‐watershed scales. The current map (Figure 2) revealed areas of higher current density and impeded current flow. Over the entire area, predicted connectivity among reaches was highest throughout the western islands of the study area. Predicted connectivity was also higher on the southern portion of the mainland. The higher current density observed in these areas indicated multiple linkages between watersheds, but also identified connectivity between multiple reaches within watersheds (Figure 2B,C). The eastern half of our study area, which is more mountainous, also displayed areas of high intra‐watershed connectivity (Figure 2D), but included less predicted connectivity between watersheds within this region.

**FIGURE 3:**
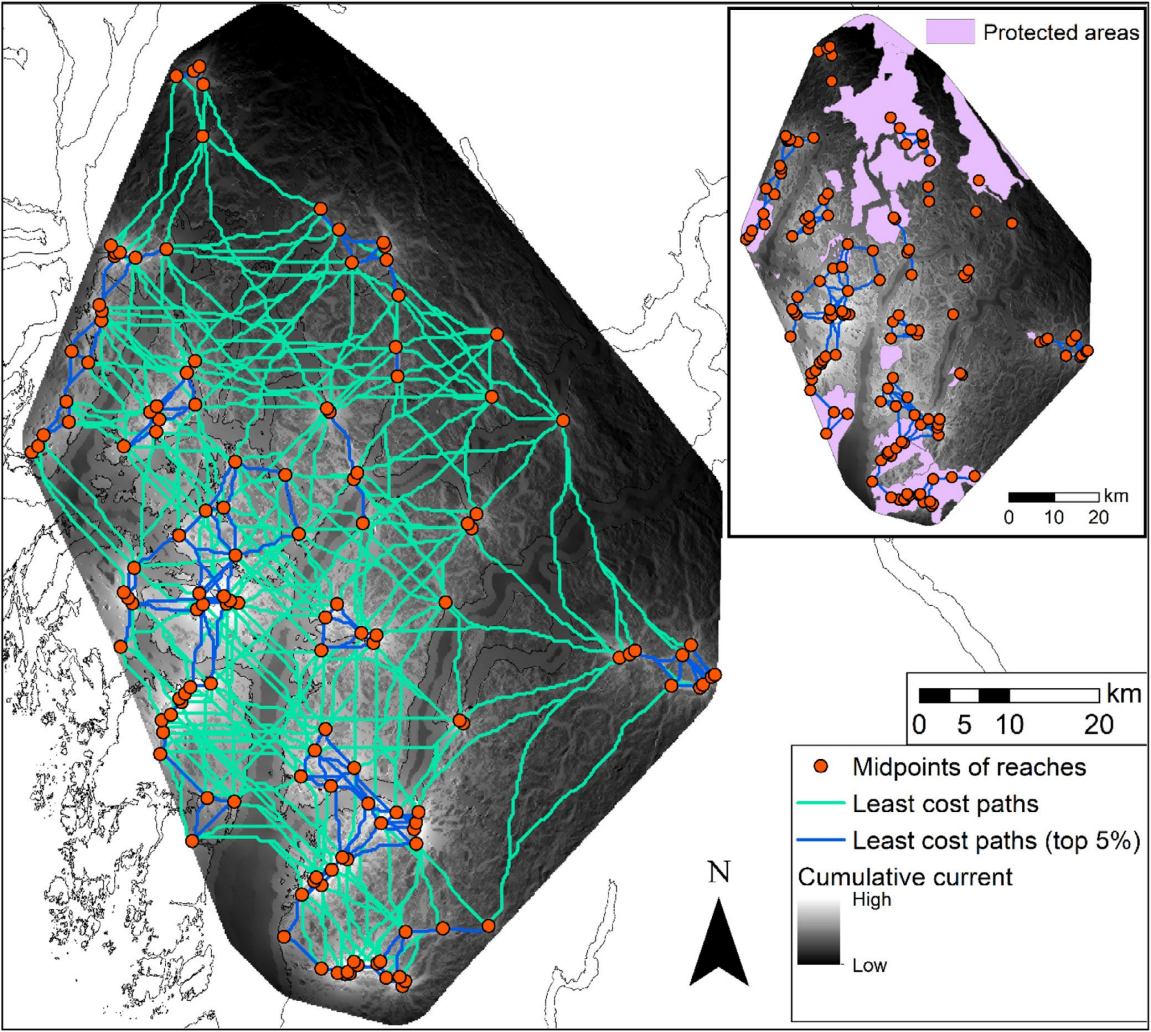
Map showing modeled least cost paths (LCP; *n* = 9045) between nodes, derived from the cumulative current layer. Approximately 65% of the top 5% LCP (with the lowest accumulated cost distances; *n* = 453) occurred outside of protected areas.

**FIGURE 4:**
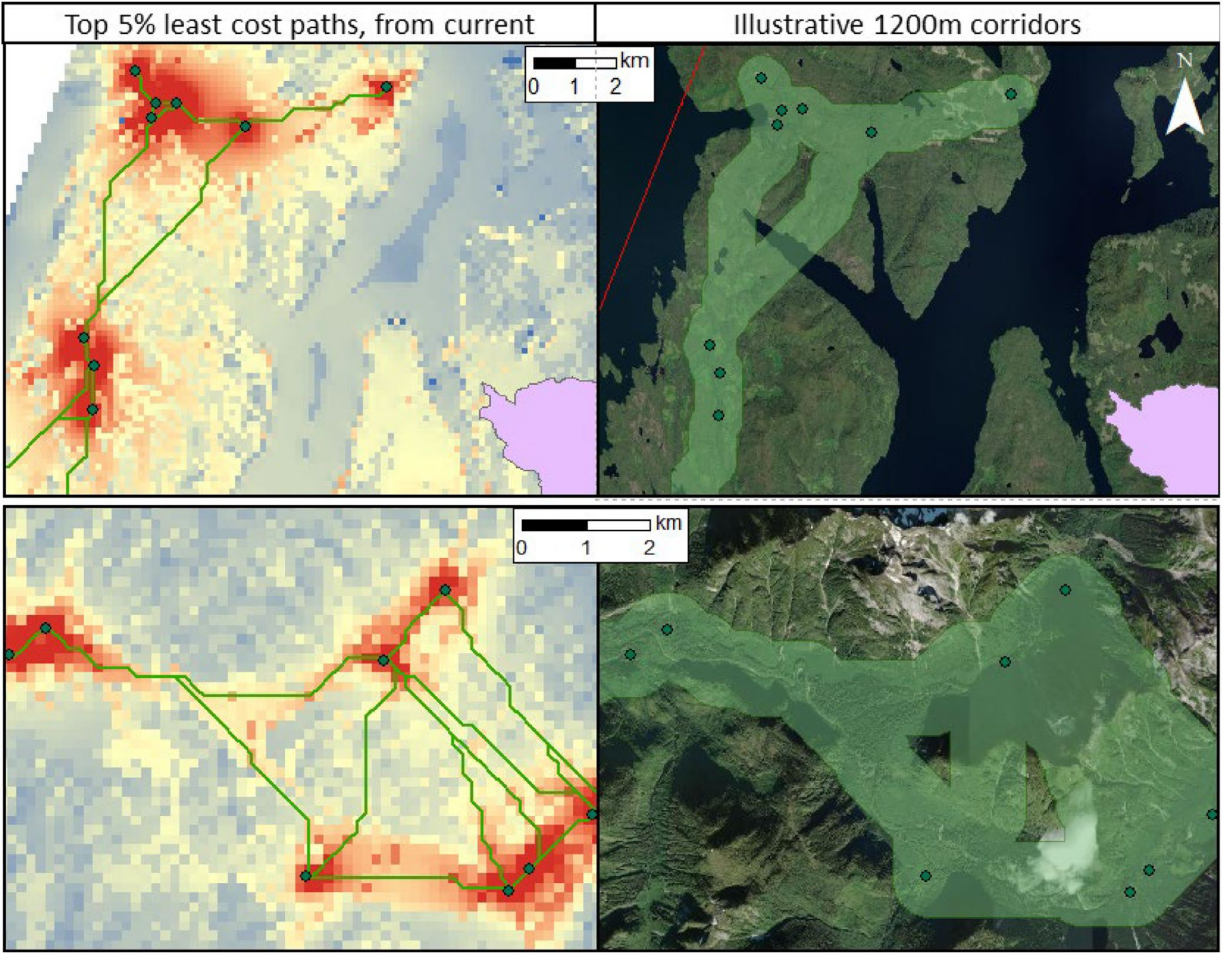
Maps illustrating example corridors between spawning salmon nodes in Haíɫzaqv (Heiltsuk) Territory on the central coast of British Columbia, Canada, based on the Circuitscape and simplified Least Cost Paths (LCP) outputs. Protected areas are shown as pink polygons. (left) We simplified the current density map output from Circuitscape (heatmap from red [high predicted movement] to blue [low predicted movement]) into a network of only the paths with highest conductance and lowest cost distances, via LCP (green lines). (right) Potential 1200 m‐wide corridors (shaded green buffers) based on the top 5% LCP.

The least cost path (LCP) analysis simplified the continuous current layer into a network of the pathways of highest conductance and lowest cost between nodes (Figure 3; *n* = 9045). From the identified LCP, we highlighted the top 5% with the lowest cost distances (e.g., the total accumulated cost) between nodes; about 65% of these top paths (422 km in accumulated length) occurred outside of protected areas. Our 1200 m width corridors (Figure 4) highlighted possibilities for connecting multiple reaches within and across watersheds, based on the top pathways.

### 3.2 Cumulative Resistance Layer Validation

**FIGURE 5:**
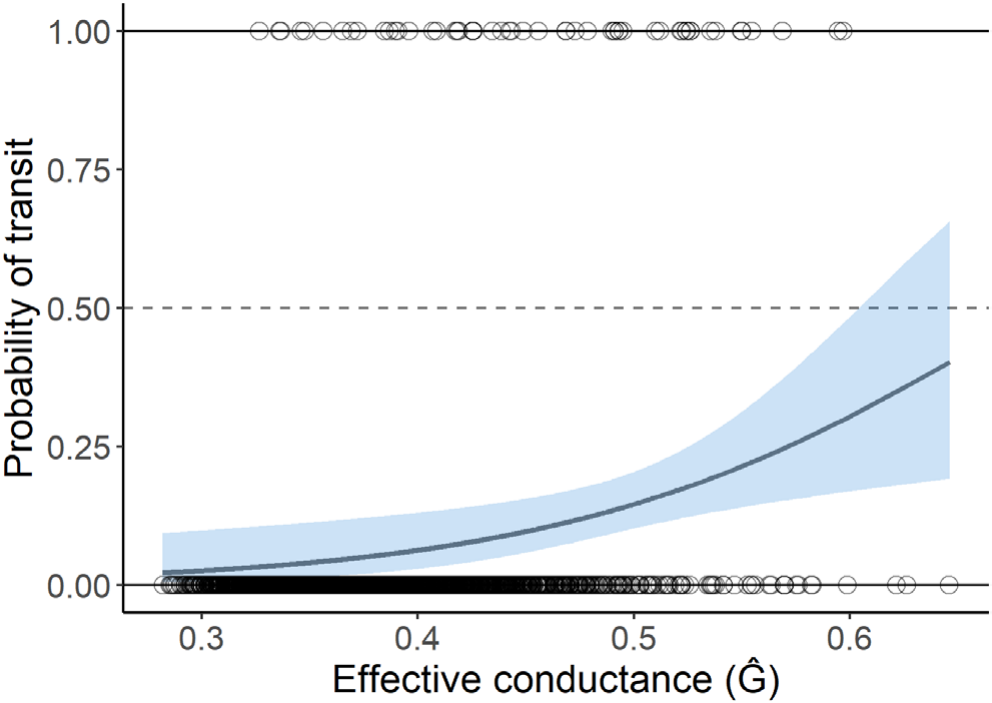
Probability of transit (binary; 0 = no transit, 1 = transit) by black and grizzly bears as a function of effective conductance (*Ĝ*) between hair snag site pairs. Points (0 [*n* = 2091], 1 [*n* = 54]) are jittered to increase visibility. Solid gray line indicates the predicted probability from the Firth's penalized‐likelihood logistic regression model and light blue shading indicate 95% confidence intervals. The Euclidean distance (m) explanatory variable is held constant at 5170 m, representing the radius of the average coastal male black bear home range (84 km^2^; Hatler et al. 2008).

**FIGURE 6:**
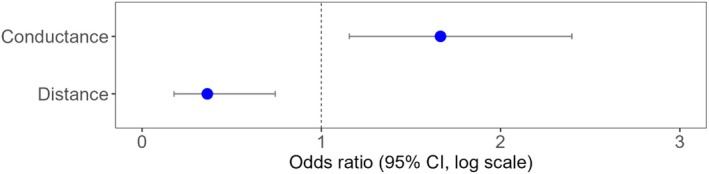
Odds ratios (blue dots) and 95% ± CI (lines) for Firth's penalized‐likelihood logistic regression model, predicting the association between effective conductance (*Ĝ*; 1.66, 1.16–2.40, *p* = 0.006) and distance (m; 0.36, 0.18–0.74, *p* = 0.006) on the probability of transit.

We found evidence to support the validation of our cumulative resistance layer. The probability of transit between snag site pairs, calculated from presence data from genetically tagged grizzly and black bear hair samples, was positively associated with the effective conductance values determined from Circuitscape (Figures 5, 6; Odds ratio = 1.66 [95% CI 1.16–2.40], *p* = 0.006). Additionally, transits were negatively associated with the Euclidean distance (m) between site pairs (Odds ratio = 0.36, [95% CI 0.18–0.74], *p* = 0.006).

## 4 Discussion

We identified potential areas of importance to grizzly and black bears for movement among salmon reaches during the spawning season. Our results from Circuitscape revealed areas of high‐current density among reaches throughout the study region, at both the inter‐ and intra‐watershed scale. Areas with the most predicted connectivity were located throughout the coastal islands and southern portion of the mainland within our study area. While the cumulative current from Circuitscape provided a range of potential pathways bears might use, the results from applying LCP modeling allowed us to further identify and rank the most optimal linkages. Overall, as the importance of phenological tracking and access to multiple spawns is well‐known for coastal bears, we believe this study provides broad landscape‐scale insight into the functional connectivity among salmon pulses. Further, as this work is applied, our results provided a generalized set of landscape planning options for HIRMD, with potential for further customization based on other objectives.

### 4.1 Functional Connectivity

We observed spatial patterns of predicted connectivity that varied across our study area. Broadly, the western islands and southern portion of the study area included the highest predicted connectivity within and across watersheds. This was not a surprise as topography is less rugged here and salmon reaches are closer to one another. By contrast, the more mountainous eastern half of the study area included areas of higher resistance, such as rugged terrain, snow, and ice. Valleys of importance were identified throughout the coast mountains via constricted areas of high‐current density, which appeared to offer the primary routes of estimated connectivity. Mountain ranges are a known impediment to bear dispersal; within the Rocky Mountains of BC and Alberta, rugged, glaciated mountains restrict gene flow in grizzly bears (Proctor et al. 2012). Accordingly, there were limited high‐current routes (depicted by Circuitscape and the narrowed‐down top 5% LCP) connecting salmon reaches across watersheds within this mountainous region (Figures 2, 3). Although natural barriers exist between eastern watersheds of our study area, local knowledge and our long‐term spring bear monitoring work (Henson et al. 2021) have previously revealed that both bear species move great distances throughout the area, even within seasons. Indeed, via genetic tagging of the hair data, individuals have previously moved between King Island (the large island located center of our study area, Figure 1) and the northern mountainous region, as well as between King Island and the southernmost tip of our study area (*unpublished data*). In other coastal areas, mean home ranges of bears are highly variable, ranging from 25 km^2^ for female black bears to at least 130 km^2^ for male grizzlies (MacHutchon et al. 1993; Sager‐Fradkin et al. 2008). Therefore, conserving high‐current connections between reaches at both inter‐ and intra‐watershed scales presents a cautious planning option to facilitate the movement of individuals over the long term.

### 4.2 Limitations of Our Analysis

As with any ecological modeling, predicting the movement of species comes with challenges and limitations. For example, most approaches for connectivity modeling require assumptions that individuals navigate either optimally (with complete knowledge of the landscape, following LCP) or randomly (with no knowledge of landscape, following circuit theory); however, such assumptions on movement preferences are potentially unrealistic (Coulon et al. 2015; Goicolea et al. 2021). Furthermore, whereas information on all possible movement probabilities across the landscape may provide important ecological information, land managers and decision‐makers often require simplified options (Parrott et al. 2019). As such, modeling approaches must balance the simplification of results for realistic application with the integration of some level of fluidity and plasticity in species' movement preferences. Our methods of providing HIRMD with the current density map from Circuitscape, in addition to the narrowed‐down network of only the least costly current density paths (similar to the skeleton network created by Parrott et al. 2019), aimed to address comprehensively the opportunities and challenges of both approaches. However, we acknowledge recent advances in connectivity modeling techniques may alternatively have worked in our case. For example, stochastic movement simulator (SMS; Palmer et al. 2011) models incorporate sequential decisions an individual may make within their perceptual range. Additionally, randomized shortest path (RSP; Panzacchi et al. 2016) allows users to parameterize a level of “randomness (*θ*)” within the model, which spans from completely random (*θ* = 0) to optimal (*θ* = ∞). Whereas such approaches allow users to adjust model parameters to better represent their study system, they increase computational load, particularly across large landscapes (Coulon et al. 2015; Van Moorter et al. 2022). Recent applications such as ConScape (Van Moorter et al. 2022), however, advance the RSP framework and show promise for future applications to efficiently integrate randomness into connectivity models over large areas (Van Moorter et al. 2023). In our case, using Circuitscape and Least Cost Paths provided the most value as a preliminary step in this applied context, and was aligned with our common goals, analytical expertise, and mutual understanding of the bear‐salmon system of the area.

Accounting for the ecological complexity and dynamics of the environment within a connectivity model poses additional challenges. Unnithan Kumar et al. (2022), for example, argued that important context‐dependent variables, such as intra‐ and interspecific influences on individual movements, are often missing in models. Within our study system and those in neighboring Alaska, evidence suggests there is competition among black and grizzly bears, as well as wolves, over salmon (Smith et al. 2004; Service et al. 2019), dynamics not accounted for in our modeling. Additionally, the habitat variables and parameters we selected for our resistance surface were generalized to grizzly and black bears and might not accurately represent the factors that affect daily movements by different age and sex classes, as well as individuals. Moreover, as our aim was to estimate functional connectivity among the diverse salmon spawns across the entire spawn season, we aggregated spatiotemporal dynamics into one static resistance surface. While this approach excluded important information on the temporal within‐season dynamics of this resource wave (e.g., changes in spawn locations and landscape features throughout the period), it was appropriate for our objectives and provided a broad spatial overview of the accessibility of salmon pulses. Further, the use of the midpoints of salmon reaches as focal nodes potentially reduced the accuracy for where the bulk of spawning biomass occurs and may not capture detailed within‐reach movements by bears. However, we used the midpoints of reaches because detailed data on precise spawning locations along reaches are not available. Additionally, given the maximum reach length of 11.3 km within our study area, our midpoints occurred within a maximum of ~ 5.6 km from potential spawning sites along a reach, which is a small distance for bears along a relatively homogenously permeable extent of stream, considering the landscape‐scale size and variation in permeability we assessed. We reason that such spatial imprecision at the scale of individual salmon reaches likely does not unduly affect the accuracy of modeled connectivity for these highly mobile species. We suspect that advancements to connectivity models will allow for the inclusion of temporal and fine‐scale spatial dynamics in future predictions for this ecological system.

The methods of parameterizing and validating our cumulative resistance surface included additional limitations. Our habitat variable and resistance parameterization decisions could not leverage telemetry or empirical movement data, and we instead estimated resistance by drawing on habitat preferences by bears as observed in the literature and by Haíɫzaqv Knowledge. Cushman et al. (2013) noted limitations to this approach, primarily that patterns of habitat preferences do not necessarily reflect the influence of landscape features on connectivity, but rather the maximization of fitness in individuals. Further, our resistance scale (1–6 for individual resistance layers; 1–10 for cumulative resistance layer) was subjective, which Sawyer and Kauffman (2011) emphasized imposes a limitation in resistance‐based connectivity modeling. Our approach for validating our cumulative resistance surface by using transits inferred from genetically identified hair samples additionally involved assumptions. For example, although we could determine whether site pairs had been transited within a sampling season based on the genetic identity of the hair collected at each, we could not determine the actual paths taken by individuals. However, it is unlikely that transits included circuitous routes taken by bears between sites, owing to the energetic costs such routes would impose and the narrow sampling windows (sites revisited twice, 10–14 days apart) within our sampling season (four to six weeks). Finally, our hair samples were collected during the spring (May and June), which is only a short window and outside of the entire salmon spawn period. However, we do not expect resistance to movement to differ throughout the year.

### 4.3 Relevance and Future Applications

Our work contributes to the growing body of connectivity modeling research on dynamic ecological systems. As landscapes face increasing pressure from human use worldwide, a better understanding of the movements and resource accessibility by mobile species is required for planning and retaining functional connectivity, particularly in environments with lower human disturbance (Parrott et al. 2019). To our knowledge, connectivity models have not yet been applied in the context of coastal bears and spawning salmon. Evidence from coastal BC and Alaska highlights the importance of a diversified and sustained salmon portfolio to both bear species throughout the spawn season (Deacy et al. 2018; Service et al. 2019). Accordingly, our results provided landscape‐scale information on the estimated connectivity among salmon spawns within and across watersheds. While our methods simplified phenological diversity into one landscape surface, future research within this system could examine using entire reaches and consider actual temporal patterns (i.e., order of salmon species spawns). Overall, however, we believe our results provide new summary understanding of the extent to which salmon spawns are currently accessible to coastal bears within this relatively intact coastal landscape.

More locally, this work provides an example of applied connectivity research co‐developed with a local government, bridging a gap often found in connectivity projects between research outputs and government planning and implementation. Indeed, a major challenge in connectivity research is effective translation of scientific findings into connectivity potential for managers (Githiru et al. 2011; Keeley et al. 2018; Gray et al. 2020). Our analysis provided general information to HIRMD on important networks for black and grizzly bears to access salmon within their Territory, as well as the extent to which these exist outside of protected areas. HIRMD's Forestry Department oversees forestry management and planning and requested information to guide landscape planning. After co‐developing the research questions, the proposed methods were shared and discussed to align with the values and management goals of HIRMD. In collaboratively setting the resistance parameters, we ensured the model was parameterized to reflect functional connectivity and bear movement within this specific region, as informed by not only the literature but also by localized knowledge. Such a “Two‐Eyed Seeing” approach, which is informed by Western scientific and Indigenous lenses, can provide more comprehensive insight than any one vantage alone (Service et al. 2014; Reid et al. 2021). Especially in the context of modeling and parameterization, a process that often requires qualitative information and/or “expert opinion”, Indigenous Knowledge systems hold detailed past and present information on environmental dynamics. Indeed, millennia of observation and science have fostered a deep and complex understanding of ecological systems, an understanding that Western science can complement. Finally, research conducted by a team that includes decision‐makers, as we have done here, makes the application of evidence much more likely. Discussion and consultation continued as results emerged and throughout the manuscript preparation process. Critically, all spatial inputs and outputs, including metadata and a technical report summarizing the project, were shared with HIRMD to support application. Geomatics products were particularly deemed important to support decision‐making in specific areas of the Territory. Further work and engagement on this project are ongoing to determine next steps. In other words, our team does not consider the project as concluding when a scientific paper is published. As in years past, one project leads to another in a continual process.

## Author Contributions

**Ilona Mihalik:** conceptualization (equal), data curation (lead), formal analysis (lead), investigation (lead), methodology (lead), project administration (lead), visualization (lead), writing – original draft (lead), writing – review and editing (equal). **Mathieu Bourbonnais:** conceptualization (equal), data curation (supporting), formal analysis (supporting), methodology (supporting), writing – original draft (supporting), writing – review and editing (equal). **William Housty:** conceptualization (equal), data curation (supporting), formal analysis (supporting), methodology (supporting), writing – review and editing (equal). **Kevin Starr:** data curation (supporting), formal analysis (supporting), methodology (supporting), writing – review and editing (equal). **Paul Paquet:** supervision (supporting), writing – review and editing (equal). **Chris Darimont:** conceptualization (equal), data curation (supporting), formal analysis (supporting), methodology (supporting), supervision (lead), writing – original draft (supporting), writing – review and editing (equal).

## Disclosure

Given the applied nature of our work, our target audience includes conservation scientists, wildlife ecologists, environmental planners, land managers, and policymakers.

## Conflicts of Interest

The authors declare no conflicts of interest.

## Data Availability

All spatial data analyzed during this study are publicly available from the BC Data Catalog at https://catalogue.data.gov.bc.ca. Annotated code is available in the project's GitHub repository at https://github.com/ilonamihalik/HT‐connectivity. Due to agreements with the Haíɫzaqv Nation, sampling locations for the genetic recapture data cannot be provided.
